# Supplementary material for: Assembly of Hollow Yttrium Oxide Spheres from Nano-Sized Yttrium Oxide for Advanced Passive Radiative Cooling Materials
Source: Polymers (Basel). 2024 Jun 9;16(12):1636. doi: 10.3390/polym16121636 (PMC11207504; doi:10.3390/polym16121636)
Supplement: Supplementary file 1 [file polymers-16-01636-s001.zip › polymers-3047925-supplementary.pdf]

Supporting information

Assembly of Hollow Yttrium Oxide Spheres from Nano-Sized Yttrium Oxide for Advanced Passive Radiative Cooling Materials

Jeehoon Yu, Daeyul Kwon, Heegyeom Jeon and Youngjae Yoo *

Department of Advanced Materials Engineering, Chung-Ang University, Anseong 17546, Republic of Korea

* E-mail: yjyoo@cau.ac.kr

Keywords: passive radiative cooling; polydimethylsiloxane; yttrium oxide; particle structure control; hollow particle; assembled nanoparticle

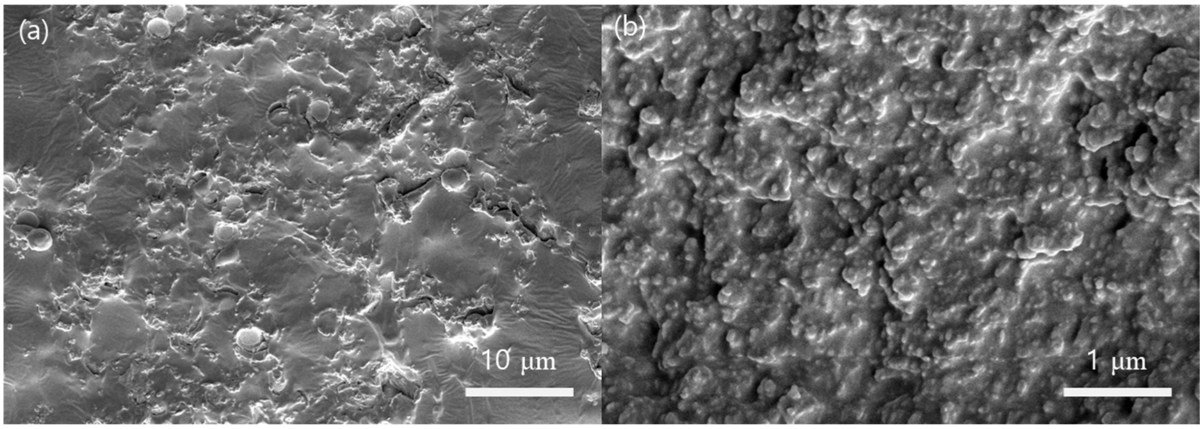

Figure S1. High-magnification cross-sectional images of AHYOSPs and NYO films.
